# Hard X‐Ray Nanotomography for 3D Analysis of Coking in Nickel‐Based Catalysts

**DOI:** 10.1002/anie.202106380

**Published:** 2021-08-31

**Authors:** Sebastian Weber, Darren Batey, Silvia Cipiccia, Matthias Stehle, Ken L. Abel, Roger Gläser, Thomas L. Sheppard

**Affiliations:** ^1^ Institute for Chemical Technology and Polymer Chemistry Karlsruhe Institute of Technology (KIT) Engesserstr. 20 76131 Karlsruhe Germany; ^2^ Institute of Catalysis Research and Technology Karlsruhe Institute of Technology (KIT) Hermann-von-Helmholtz-Platz 1 76344 Eggenstein-Leopoldshafen Germany; ^3^ Diamond Light Source Harwell Science and Innovation Campus Fermi Ave Didcot OX11 0DE UK; ^4^ Dept. of Medical Physics & Biomedical Engineering University College London Malet Place, Gower Street London WC1E 6BT UK; ^5^ Institute of Chemical Technology Universität Leipzig Linnéstr. 3 04103 Leipzig Germany

**Keywords:** carbon, methanation of CO_2_, nickel, Raman spectroscopy, X-ray ptychography

## Abstract

Understanding catalyst deactivation by coking is crucial for knowledge‐based catalyst and process design in reactions with carbonaceous species. Post‐mortem analysis of catalyst coking is often performed by bulk characterization methods. Here, hard X‐ray ptychographic computed tomography (PXCT) was used to study Ni/Al_2_O_3_ catalysts for CO_2_ methanation and CH_4_ dry reforming after artificial coking treatment. PXCT generated quantitative 3D maps of local electron density at ca. 80 nm resolution, allowing to visualize and evaluate the severity of coking in entire catalyst particles of ca. 40 μm diameter. Coking was primarily revealed in the nanoporous solid, which was not detectable in resolved macropores. Coke formation was independently confirmed by *operando* Raman spectroscopy. PXCT is highlighted as an emerging characterization tool for nanoscale identification, co‐localization, and potentially quantification of deactivation phenomena in 3D space within entire catalyst particles.

## Graphical abstract

The formation of coke in nickel‐based catalyst particles can be investigated by ptychographic X‐ray computed tomography with sub‐100 nm resolution. Based on localized changes of electron density between the activated and coked catalysts, the location of coke can be resolved in 3D at the nanoscale. This approach can be used to study different catalyst deactivation pathways on multiple length scales from nm to μm.

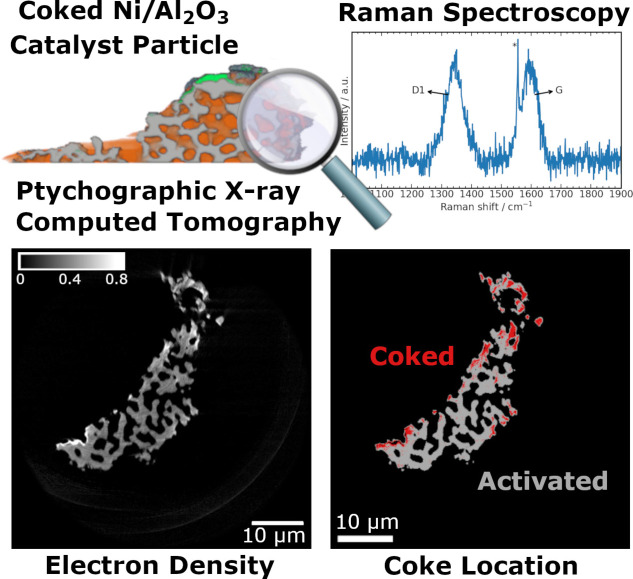

Understanding deactivation mechanisms in heterogeneous catalysis is of fundamental importance for both industrial and academic research, in terms of improving catalyst stability and performance. A common catalyst deactivation route in reactions involving carbon‐containing species is formation and deposition of different solid carbonaceous species on the catalyst. This is often referred to as coking, and may lead to covering of active sites or blocking of pores for example.[^1^, ^2^, ^3^, ^4^, ^5^] Dry reforming of CH_4_ and methanation of CO_2_ are two reactions utilizing CO_2_ as feedstock typically performed over supported Ni‐based catalysts.[^6^, ^7^, ^8^, ^9^] For dry reforming of CH_4_, coke formation is a major deactivation route,[^6^, ^7^] and it is also discussed as a possible deactivation route for methanation catalysts.[^8^, ^9^, ^10^] Tailoring the catalyst pore system by introducing hierarchical meso‐ (2–50 nm) and macropores (>50 nm) has been suggested to improve catalyst stability and performance. Specifically, introducing larger pores for molecular transport or diffusion can help mitigate pore blocking.[^11^, ^12^] However, to evaluate optimized pore systems and promote rational design for coke suppression, a detailed spatially‐resolved understanding of coke formation is required.

Commonly applied methods to investigate catalyst coking mainly retrieve bulk information, such as amount and elemental composition of coke.[^4^, ^5^] This is partly due to the difficulty in separating or distinguishing different coke species on the solid catalyst. On the other hand, with microscopy or other 2D spatially‐resolved characterization methods (e.g. FTIR or Raman spectroscopy), no separation is needed and coke can be studied directly on the catalyst surface. There are only few techniques reported to extend spatially‐resolved characterization to 3D space, which can provide a more representative view of sample structure. These include electron microscopy (EM),[^13^, ^14^] atom probe tomography (APT)^[15]^ and soft X‐ray scanning transmission microscopy (STXM).^[16]^ A main drawback of the above methods is that they only cover limited length scales, while coking is a relevant phenomenon from the mesopore up to the whole particle or even the reactor scale. Ideally, high spatial resolution over extended sample length scales should be combined in a single measurement, or complementary methods should be used to include information from different scales.^[17]^ This is especially important for materials with hierarchical meso‐ and macropore systems (or other heterogeneous samples), where the pore system itself ranges from nm to μm and the catalyst particles may range from μm to cm. EM and APT provide high‐resolution data in the nm range, but on limited and localized sample volumes. STXM can cover nm to μm and can directly provide information about the carbon species by X‐ray absorption spectroscopy at the C‐K‐edge, but with soft X‐rays this is limited to relatively thin samples due to strong attenuation of the transmitted beam. Hard X‐ray imaging techniques can overcome those limitations and arguably have the greatest potential for high resolution imaging of large samples. One developing method known as ptychography, and its 3D counterpart ptychographic X‐ray computed tomography (PXCT), enable quantitative measurement of the sample's local electron density (*N_e_*) in 2D or 3D space.[^18^, ^19^, ^20^, ^21^] An iterative algorithm retrieves the phase shift induced by interaction between X‐rays and sample, from which the 3D *N_e_* can be directly calculated even without a priori knowledge.[^18^, ^19^, ^20^, ^22^] *N_e_* from PXCT provides chemical information about the sample, for example, location of pores, zeolite and clay within fluid catalytic cracking (FCC) catalysts.[^23^, ^24^] In addition to chemical information, PXCT offers exceptional spatial resolution among hard X‐ray methods, so far reaching 10 nm on samples up to several μm in size.[^20^, ^25^] Recently, X‐ray holotomography was also reported to retrieve coke location within 60 μm diameter FCC particles at ca. 200 nm resolution. However, the differential contrast approach shown was sensitive to relative changes in *N_e_* in an arbitrary unit scale, without providing a physical unit for *N_e_*. Furthermore, PXCT greatly exceeds the maximum resolution so far shown with holotomography.^[26]^

Here, we demonstrate the potential of PXCT to analyze coking of Ni/Al_2_O_3_ catalysts in 3D, with potential uses in CO_2_ methanation and CH_4_ dry reforming. The catalysts were prepared via two different routes, resulting in a purely mesoporous material (Ni/Al_2_O_3_‐m) by a homogenous deposition‐precipitation method^[27]^ and a hierarchical meso‐/macroporous material (Ni/Al_2_O_3_‐h) via a modified sol‐gel synthesis.[^17^, ^28^] The porosity of the samples was analyzed in previous studies and presence of macropores in the range from 50 to 100 nm is excluded based on those results.[^17^, ^27^] Both catalysts have a similar Ni loading of 17 wt. % for Ni/Al_2_O_3_‐m and 18 wt. % for Ni/Al_2_O_3_‐h. The samples were treated in a quartz capillary setup to obtain an activated and artificially coked sample of each, as illustrated in Scheme 1 (see Supporting Information for further details). Briefly, all samples were first activated in 25 % H_2_/He (${\dot{V}}_{tot}$ =20 mL min^−1^), followed by 30 min of CO_2_ methanation reaction conditions 20 % H_2_/5 % CO_2_/He (${\dot{V}}_{tot}$ =20 mL min^−1^, 673 K), resulting in the activated catalyst samples Ni/Al_2_O_3_‐ma and Ni/Al_2_O_3_‐ha (Figure S1–2). Catalytic activity was quantified during this process by online mass spectrometry (see Supporting Information). In a separate experiment, artificial coking conditions of 4 % CH_4_/ He (${\dot{V}}_{tot}$ =20 mL min^−1^, 673 K, 30 min) were then applied, similar as reported by Mutz et al.^[29]^ Treatment of artificially coked samples Ni/Al_2_O_3_‐mc and Ni/Al_2_O_3_‐hc was monitored by *operando* Raman spectroscopy, confirming the formation of carbonaceous species.

**Scheme 1:**
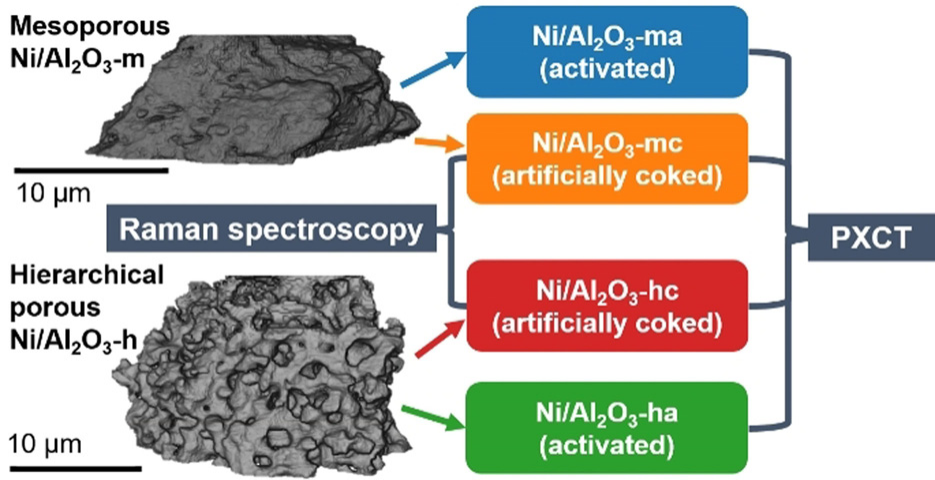
Experiments to study coke formation on two Ni/Al_2_O_3_ catalysts with different pore structures. Treatments consisted of activation, reaction conditions and additional artificial coking (see Supporting Information). The coking was followed by operando Raman spectroscopy. All samples were then investigated by PXCT.

Figure 1 shows online mass spectrometry data and temperature profiles for the artificially coked samples together with 2D plots of the *operando* Raman spectra. Neither sample showed significant Raman bands before artificial coking. Upon artificial coking, formation of typical carbon bands was observed and assigned to carbonaceous species for Ni/Al_2_O_3_‐mc (Figure 1 b,c) and Ni/Al_2_O_3_‐hc (Figure 1 e,f). The D1 band (≈1340 cm^−1^) originating from the vibration of disordered graphitic lattice (A_1g_ symmetry), and the G band originating from ideal graphitic lattice vibrations with E_2g_ symmetry (≈1590 cm^−1^) were detected.[^29^, ^30^, ^31^] The presence of carbonaceous species was therefore confirmed by *operando* Raman spectroscopy for both Ni/Al_2_O_3_‐mc and Ni/Al_2_O_3_‐hc, but as a bulk characterization this provides limited information about the location of coke species in the particles.

**Figure 1:**
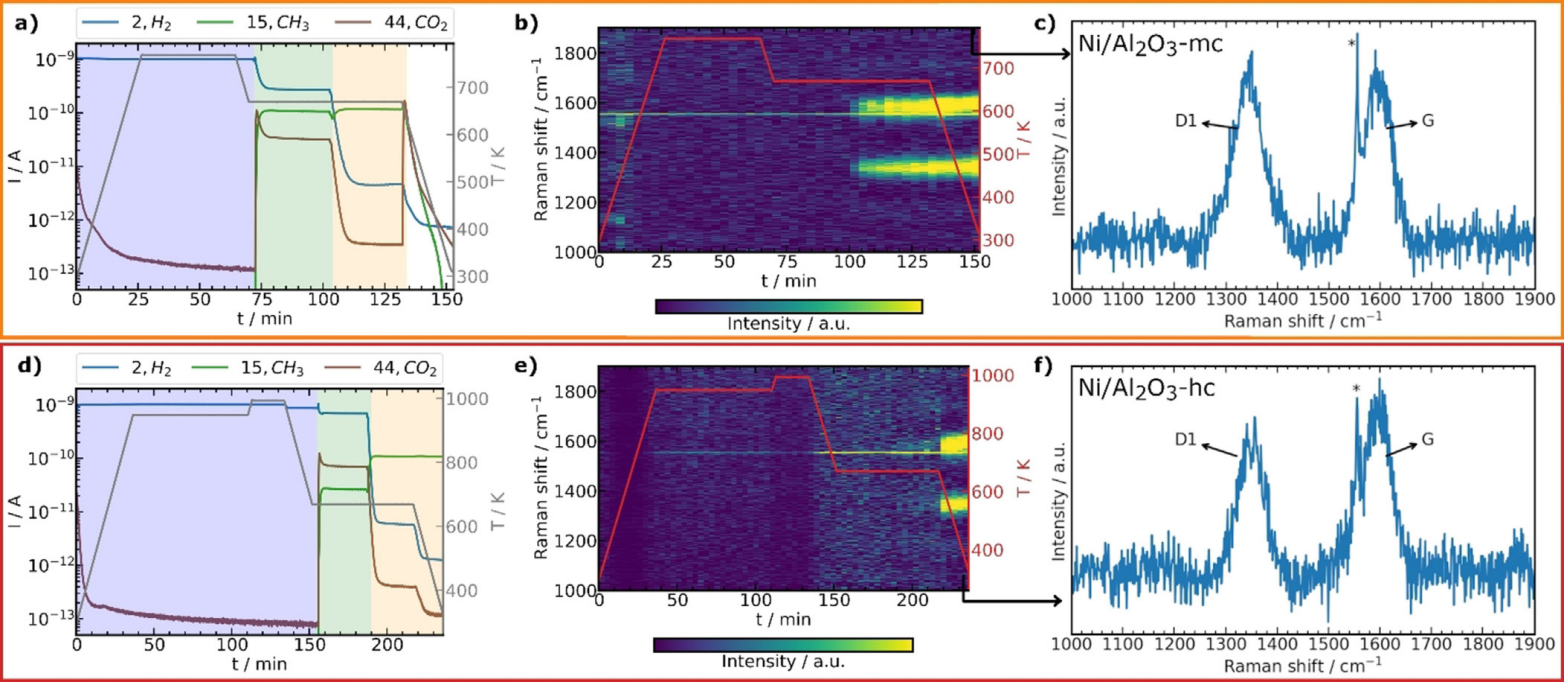
Operando Raman spectroscopy of Ni/Al_2_O_3_‐mc (a–c) and Ni/Al_2_O_3_‐hc (d–f). a,d) selected mass spectrometry traces during activation (blue), reaction (green) and artificial coking (orange) with temperature (*T*, gray); cooling in He (white). b,e) 2D plots of Raman spectra (2× binned) with the last spectra highlighted in (c,f) showing formation of carbon (D1 and G bands). * Line feature at 1557 cm^−1^ in (b,c,e,f) corresponds to O_2_ from air.^[32]^

Samples Ni/Al_2_O_3_‐ma, Ni/Al_2_O_3_‐mc, Ni/Al_2_O_3_‐ha and Ni/Al_2_O_3_‐hc were then investigated by PXCT to study the distribution of carbonaceous species within the catalyst particles. From catalyst powders obtained after treatment, suitable particles of each sample with diameters of about 30 to 50 μm were selected and mounted on Al tomography pins using a dual‐beam focused ion beam scanning electron microscope (FIB‐SEM) (Figure S3,4). PXCT experiments were carried out at I13‐1 beamline of the Diamond Light Source (Oxford, UK)^[33]^ to obtain 2D ptychography projections using 400 iteration of the PtyREX package^[34]^ based on the ePIE algorithm^[35]^ (see Supporting Information, Table S1). The tomograms were aligned and reconstructed using the script of Odstrčil et al.^[36]^ with a resulting voxel size of ca. 37 nm for all samples. The effective resolution of the tomograms was estimated by Fourier shell correlation as between 74 to 83 nm (Figure S5–8). The *N_e_* tomograms were calculated from the reconstructed complex refractive index *δ*(r) tomograms (see Supporting Information).[^18^, ^19^] Thus, 3D quantitative *N_e_* distribution in the catalyst particles was obtained for Ni/Al_2_O_3_‐h (Figure 2) and Ni/Al_2_O_3_‐m (Figure S10) samples. The results for the Ni/Al_2_O_3_‐m samples are presented and discussed in the Supporting Information. Especially for the Ni/Al_2_O_3_‐mc sample some phase artifacts could not be resolved, which may influence the projection alignment and obtained *N_e_* values, therefore limiting the reliability of the coking analysis. This issue did not affect the Ni/Al_2_O_3_‐h data which is discussed below. For *N_e_* analysis, the particles were first masked from the surrounding air to obtain a label of the whole catalyst particle. The obtained particle label was further segmented by thresholding into pores (orange), catalyst body (gray) and contamination (green) labels (Figure 2, Figure S10–12,17,18). The contamination originates from Pt used during FIB preparation of the samples on the tomography pins, which can be easily segmented due to relatively high *N_e_* and discarded from further analysis. One can clearly observe the different pore structures of the two systems. For Ni/Al_2_O_3_‐h (Figure 2) a connected macropore network is observed, while for Ni/Al_2_O_3_‐m (Figure S10) only few voids within the particles are present. In Ni/Al_2_O_3_‐hc no clear coke formation inside the macropores or voids was detected (Figure 2 b).

**Figure 2:**
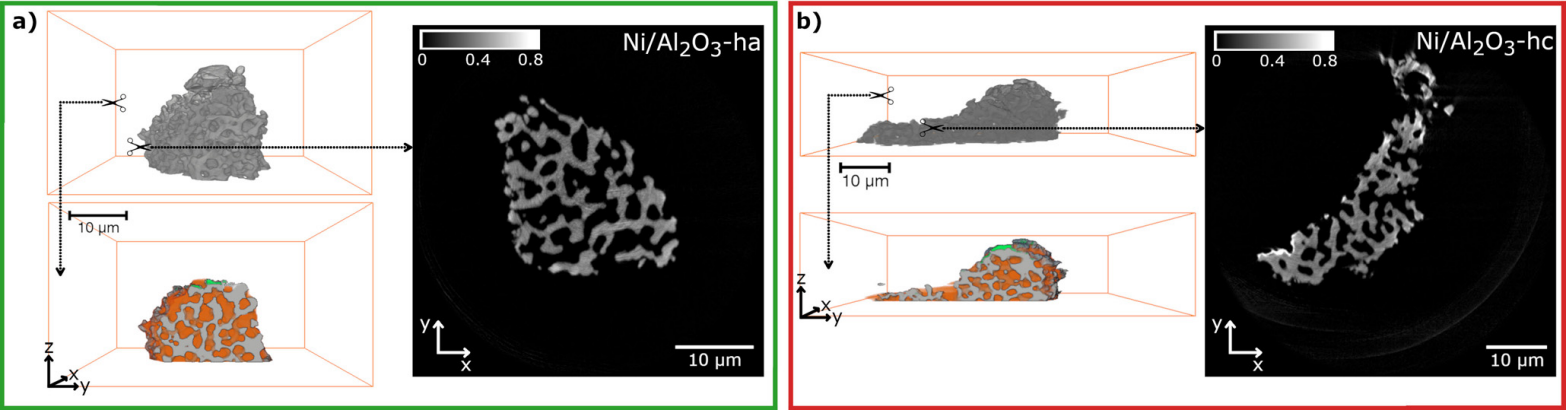
PXCT of activated catalyst Ni/Al_2_O_3_‐ha (a) and artificially coked catalyst Ni/Al_2_O_3_‐hc (b). Each reconstructed volume (gray) is shown with a cut through the middle, illustrating the segmented and labeled tomograms (gray=nanoporous catalyst body, orange=pores, green=contamination) and a grayscale *N_e_* image of a typical slice through the volume (color bar in *N_e_* / e^−^ Å^−3^, *N_e_* offset to 0 with respect to air close to the sample, see Supporting Information).

While the resolution obtained (ca. 80 nm) is not sufficient to directly visualize the presence and location of coke by thresholding and image segmentation, this is a common limitation of all current hard X‐ray microscopy methods. However, PXCT as shown here has the advantage of providing a quantitative measure of the local *N_e_* distribution. This property can be used to objectively evaluate coke formation in terms of variation in *N_e_* within the sample, but is in principle additionally sensitive to sample elemental composition, bonding, atomic configuration, and other fine structural variations. Figure 3 a shows the global *N_e_* distribution within whole particles of Ni/Al_2_O_3_‐h. Prior to analysis, *N_e_* was offset to 0 with respect to air in close proximity to the sample (for analysis method see Supporting Information). A peak around 0.0 e^−^ Å^−3^ was assigned to air in the macropores, while a second peak with maxima around 0.4 e^−^ Å^−3^ corresponds to the nanoporous catalyst body, and peaks at higher *N_e_* values to Pt contamination. In the case of Ni/Al_2_O_3_‐hc (Figure 3 a) no clear shift of the *N_e_* mode for the artificially coked sample was detected compared to the Ni/Al_2_O_3_‐ha sample. However, the appearance of a slight shoulder at higher *N_e_* values can be observed as highlighted in the normalized *N_e_* of the catalyst body labels (Figure S23). As operando Raman spectroscopy revealed clear evidence of coking under the applied conditions, we therefore assign the appearing shoulder at increased *N_e_* to coke formation within the nanoporous catalyst body of Ni/Al_2_O_3_‐hc. We note that in principle, *N_e_* is sensitive to any variation of local chemical structure or bond/atom arrangement. This constitutes a powerful characterization potential, but also requires caution in interpretation. Relative increases in *N_e_* may result not only from coke‐filled pores, but also effects such as pore collapse, sintering of active metal, crystallographic changes in catalyst support, or other such effects. Since the treatments of Ni/Al_2_O_3_‐ha and Ni/Al_2_O_3_‐hc performed in the capillary setup differ only by the artificial coking step, which is lower in temperature than that of the initial activation, we can therefore reasonably conclude that changes in *N_e_* observed are most likely a result of coking.

**Figure 3:**
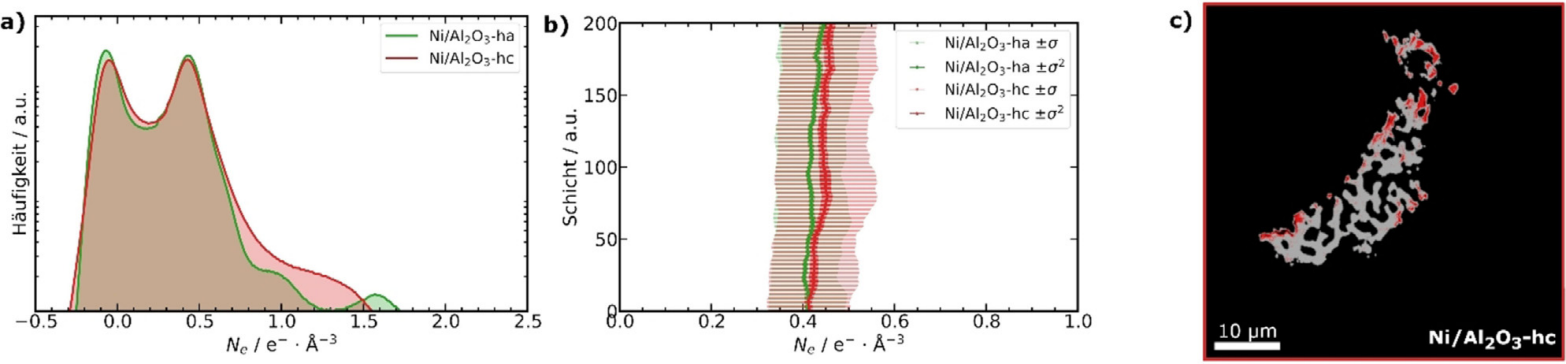
Global electron density distribution from PXCT of the activated and artificially coked catalyst particle labels of Ni/Al_2_O_3_‐h (a). Mean, standard deviation (*σ*) and variance (*σ* ^2^) in *N_e_* of the segmented catalyst body label in a selected slice range (b). Segmented tomogram slice of Ni/Al_2_O_3_‐hc (c) showing less electron dense (gray, assigned to activated catalyst body) and higher density (red, assigned to coked catalysts body) regions by binary thresholding of the normalized *N_e_* distribution (Figure S21). The reliability of the thresholding‐based segmentation was tested for different thresholds (Figure S23).

For finer spatially‐resolved statistical analysis of the tomography data, we first checked the dependence of the mean *N_e_* of the whole particle label on specific tomogram slices (Figure S22). Regions with strong Pt contamination are easily identified and excluded, so further analysis was limited to slices 1 to 200. The slice‐dependent analysis of the mean *N_e_* of the Ni/Al_2_O_3_‐h samples shows an increase of the *N_e_* for the coked sample (Figure 3 b, see Supporting Information Table S3). The mode of the *N_e_* is almost identical for the Ni/Al_2_O_3_‐ha and Ni/Al_2_O_3_‐hc samples (Figure S24), but the standard deviation (*σ*) clearly shows the increased contribution mainly from higher *N_e_* regions for Ni/Al_2_O_3_‐hc and thus significant increase of *N_e_* (Figure 3 b, Figure S24).

As the slice‐dependent analysis only provides information along the *z*‐direction of the tomograms, we further segmented the tomograms based on the normalized *N_e_* distribution of the catalyst body labels to estimate the location of more prevalent coking (Figure S23). For thresholding‐based segmentation we chose a clearly recognizable feature. The point of the normalized *N_e_* distribution of the catalyst body label from which Ni/Al_2_O_3_‐hc differed from Ni/Al_2_O_3_‐ha (Figure S23) was selected. The resulting further segmented catalyst body labels for Ni/Al_2_O_3_‐hc are shown in Figure 3 c, separated into an activated and coked catalyst body label based on the chosen thresholds (Table S3). It can be observed that the coke label is stronger distributed at the exterior of the particle, from which we postulate that coking may start in mesopores at the exterior of the catalyst particles and further progress to the center with increasing reaction time. This is furthermore supported by comparing multiple higher thresholds for the activated and coked samples (see Supporting Information, Figure S25). It should be noted that this comparison is of qualitative nature based on the *N_e_* and the resolution is not high enough to clearly identify single voxels of coking. A similar gradient of the *N_e_* during coke formation was also found in FCC catalysts particles studied with X‐ray holotomography.^[26]^ However, further detailed experiments at different coking stages are required to validate this conclusion.

The same analysis was also performed for the Ni/Al_2_O_3_‐m system as reported in the Supporting Information. A similar trend was found for the PXCT of the Ni/Al_2_O_3_‐m samples, with an even more pronounced shift of the *N_e_* upon coking (Figure S13‐18). However, due to existing phase vortices artifacts, especially in Ni/Al_2_O_3_‐mc, that might influence the projection alignment and *N_e_* values, those results should not be regarded as conclusive but rather only as indicative. The artifacts in question may occur due to distinct edge like or strong absorbing features in the sample and are a general challenge in ptychographic reconstruction of thick samples. Recently, an algorithm called “VortRem” was reported to remove such artifacts caused by non‐convergence of the ptychographic reconstruction.^[37]^ This readily shows potential limitations of PXCT depending on the studied samples, and the requirement of a careful sample selection and preparation in future studies.

In a crucial advantage over bulk measurements, PXCT can not only characterize, but directly visualize the results of deactivation phenomena with sub‐100 nm resolution in 3D space within the catalyst particle. By the nature of *N_e_*, which is in principle unique to each distinct configuration of atoms in 3D space, chemical distinction of coke species depending on the *N_e_* as suggested by Vesely et al. may be possible in future, since graphitic coke or activated carbon exhibit different *N_e_*.^[26]^ However, for such a distinction to be accurate higher spatial resolution is indispensable to more precisely assign *N_e_* within the 3D sample structure. For this reason, PXCT is positioned as the ideal technique to drive catalysis research forward, having shown astonishing progress during only a decade since its invention.^[18]^ As to the best of our knowledge no database of *N_e_* of different coke species within pores of catalysts exists yet, we believe that the creation of such is required in future as a methodological step towards truly quantitative 3D imaging of coked catalyst bodies.

In summary, PXCT is clearly emerging as the leading light among current hard X‐ray microscopy methods due to its unprecedented spatial resolution (here ca. 80 nm) and ability to recover quantitative *N_e_* values. Spatially‐resolved analysis of coking in catalyst particles is now possible with resolution below 100 nm. With the current transition towards more powerful diffraction‐limited synchrotron radiation sources, progress in experimental setups (hardware), reconstruction algorithms (software), and other factors, we expect that the achievable resolution of PXCT can be improved below 10 nm, while retaining sample sizes which can only feasibly be studied with hard X‐ray microscopy.[^20^, ^38^] Increasing resolution further allows an improved distinction of overlapping features in *N_e_* distribution and thus quantitative labeling. This enables PXCT as a unique technique to study catalyst deactivation phenomena at high resolution, producing textural and chemical information and thus having the potential to deliver advanced fundamental understanding of deactivation processes in catalysis.

## Conflict of interest

The authors declare no conflict of interest.

## Supporting information

As a service to our authors and readers, this journal provides supporting information supplied by the authors. Such materials are peer reviewed and may be re‐organized for online delivery, but are not copy‐edited or typeset. Technical support issues arising from supporting information (other than missing files) should be addressed to the authors.

Supporting Information
