# Supplementary material for: Hard X‐Ray Nanotomography for 3D Analysis of Coking in Nickel‐Based Catalysts
Source: Angew Chem Int Ed Engl. 2021 Aug 31;60(40):21772–7. doi: 10.1002/anie.202106380 (PMC8518723; doi:10.1002/anie.202106380)
Supplement: Supplementary file 1 — Supporting Information [file ANIE-60-21772-s001.pdf]

Angewandte Chemie
Eine Zeitschrift der Gesellschaft Deutscher Chemiker

Supporting Information

Hard X-Ray Nanotomography for 3D Analysis of Coking in Nickel-Based Catalysts

*Sebastian Weber, Darren Batey, Silvia Cipiccia, Matthias Stehle, Ken L. Abel, Roger Gläser, and Thomas L. Sheppard**

anie_202106380_sm_miscellaneous_information.pdf

Author Contributions

S.W. Conceptualization: Supporting; Data curation: Lead; Formal analysis: Lead; Investigation: Equal; Methodology: Equal; Project administration: Supporting; Software: Lead; Validation: Equal; Visualization: Lead; Writing—original draft: Lead; Writing—review & editing: Lead
D.B. Data curation: Supporting; Formal analysis: Supporting; Investigation: Equal; Methodology: Equal; Software: Equal; Validation: Supporting; Writing—review & editing: Supporting
S.C. Data curation: Supporting; Formal analysis: Supporting; Investigation: Equal; Methodology: Equal; Software: Equal; Validation: Supporting; Writing—review & editing: Supporting
M.S. Formal analysis: Supporting; Investigation: Supporting; Methodology: Supporting; Validation: Supporting; Visualization: Supporting; Writing—review & editing: Supporting
K.A. Formal analysis: Supporting; Investigation: Supporting; Methodology: Supporting; Validation: Supporting; Writing—review & editing: Supporting
R.G. Conceptualization: Supporting; Funding acquisition: Equal; Methodology: Supporting; Project administration: Supporting; Supervision: Equal; Validation: Supporting; Writing—review & editing: Supporting
T.S. Conceptualization: Lead; Formal analysis: Supporting; Funding acquisition: Lead; Investigation: Supporting; Methodology: Lead; Project administration: Lead; Supervision: Lead; Validation: Supporting; Visualization: Supporting; Writing—original draft: Supporting; Writing—review & editing: Equal.

Content

1. Sample Preparation and *Operando* Experiments 2
  1.1. Catalyst Synthesis 2
  1.2. Capillary Reactor and Raman Spectroscopy Experiments 2
2. Ptychographic X-ray Computed Tomography Studies 4
  2.1. Focused Ion Beam (FIB) Sample Preparation 4
  2.2. X-ray Ptychography Experiments 5
  2.3. Tomographic Reconstruction 6
  2.4. Electron Density Reference 9
  2.5. Image Analysis 11
References 18

1. Sample Preparation and *Operando* Experiments

Four different samples are discussed in this study. They are based on a mesoporous 17 wt.% Ni/Al_2O_3 catalyst prepared via a homogenous deposition-precipitation method[1] and a hierarchical porous 18 wt.% Ni/Al_2O_3 catalyst with meso- and macropores prepared via a modified sol-gel method.[2,3] In total, four different samples as following are studied, with sample abbreviation in bold:

- Mesoporous activated catalyst 17 wt.% **Ni/Al_2O_3-ma**
- Mesoporous artificially coked catalyst 17 wt.% **Ni/Al_2O_3-mc**
- Hierarchical porous activated catalyst 18 wt.% **Ni/Al_2O_3-ha**
- Hierarchical porous artificially coked catalyst 18 wt.% **Ni/Al_2O_3-hc**

The synthesis of the catalysts is briefly described in the following section 1.1. The synthesized catalysts are treated in a microreactor setup as described in section 1.2 to obtain the activated samples Ni/Al_2O_3-ma, Ni/Al_2O_3-ha and the artificially coked samples Ni/Al_2O_3-mc, Ni/Al_2O_3-hc.

1.1. Catalyst Synthesis

The initial catalysts used in this study are prepared via two different literature procedures. The mesoporous sample is obtained by homogeneous deposition-precipitation as described in detail by Mutz *et al.*[1] The hierarchical porous catalyst was synthesized via a modified sol-gel method by Herwig *et al.*[3] as described in detail in Weber *et al.*[2] Both catalyst systems were used as calcined catalysts obtained after synthesis.

1.2. Capillary Reactor and Raman Spectroscopy Experiments

The calcined catalyst samples were treated in a capillary microreactor setup suitable to perform *in situ* and *operando* Raman spectroscopy studies.[4] The setup consisted of a custom made gas-dosing system of mass flow controllers (Bronkhorst), a hot-air blower (LE MINI SENSOR KIT, Leister Technologies) and online gas analysis via an OMNI-Star GSD 320 mass spectrometer (Pfeiffer Vacuum). Fast switching between different gas compositions was achieved by a 4-way valve (VICI). Before the experiments, a temperature calibration was carried out using a type K thermocouple, placed inside an empty capillary. For the experiments, generally 4 to 5 mg of the calcined sample (100 to 200 µm sieve fraction) were placed into 1.5 mm quartz glass capillaries (Wjm - Glas Müller GmbH) and fixed with quartz wool resulting in a bed length of about 7 mm. A constant total gas flow of 20 mL min^{-1} and atmospheric pressure was used in the experiments. All catalysts were first activated (blue area) and shortly tested under reaction conditions (green area) as shown in Figure 1 (main article) and Figure S1 and Figure S2. The activated samples Ni/Al_2O_3-ma and Ni/Al_2O_3-ha were obtained after reaction conditions. The coked samples Ni/Al_2O_3-mc and Ni/Al_2O_3-hc were subsequently treated with an artificial coking procedure (orange area, cf. Figure 1, main article). The reaction and artificial coking conditions were exactly the same for both catalyst systems, while the activation procedure differed, because a higher temperature for Ni reduction is required for the Ni/Al_2O_3-h system due to incorporation of Ni into the Al_2O_3 structure.[2]

The Ni/Al_2O_3-m samples were activated by heating to 773 K catalyst bed temperature with a ramp of 20 K min^{-1} in 25 % H_2 / 75 % He (v/v). Subsequently, the reactor was cooled to 673 K with 20 K min^{-1} under activation conditions before switching to reaction conditions. The Ni/Al_2O_3-h were activated by heating to 973 K with a ramp of 20 K min^{-1} in 25 % H_2 / 75 % He (v/v) with a subsequent heating to 1023 K and cooling to 673 K with 20 K min^{-1}, before switching to reaction conditions. For both systems, reaction conditions of 20 % H_2 / 5 % CO_2 / 75 % He (v/v) were applied for 30 min at 673 K. The artificial coking conditions for Ni/Al_2O_3-mc and Ni/Al_2O_3-hc were 4 % CH_4 / 96 % He (v/v) at 673 K for 30 min similar to a previous study by Mutz *et al.*[5] The selected online mass spectrometry traces corresponding to H_2^+ (m/z = 2), CH_3^+ (m/z = 15) and CO_2^+ (m/z = 44) and the temperature for the coked

and activated samples are shown in Figure 1 (main article) and Figure S1 and Figure S2, respectively. From the mass spectrometry data of the activated samples, we estimated a CO_2 conversion during reaction conditions, based on the baseline after cooling in reaction gas mixture and the intensity during reaction conditions as shown in the following equation:

$$X_{CO_2} = \left(1 - \frac{I_{CO_2}, reaction}{I_{CO_2}, baseline}\right) \cdot 100\% \quad (1)$$

For the artificially coked samples during the described experiments, *in situ* / *operando* Raman spectroscopy was performed. An inVia Raman spectrometer (Renishaw) with a frequency doubled Nd:YAG laser (532 nm, about 100 mW laser power at the source) was used. The laser beam was focused on the capillary by a video and optical fiber probe (Renishaw) with a long working distance objective. Spectra were acquired with 50 % laser power over a range of 100-2000 cm^{-1} with a 2400 lines mm^{-1} grating and an acquisition time of 10 s per spectra. Raman spectra were analyzed using WiRE 4.4 software (Renishaw). The data treatment consisted of cosmic ray removal, truncation, and baseline subtraction, while for sample Ni/Al_2O_3-hc a normalization was applied after cosmic ray removal to reduce the influence of the strong fluorescence effects observed at high temperatures. The data was averaged by binning two spectra to create the 2D heat maps shown in Figure 1 (main article) with the last spectra shown as an example.

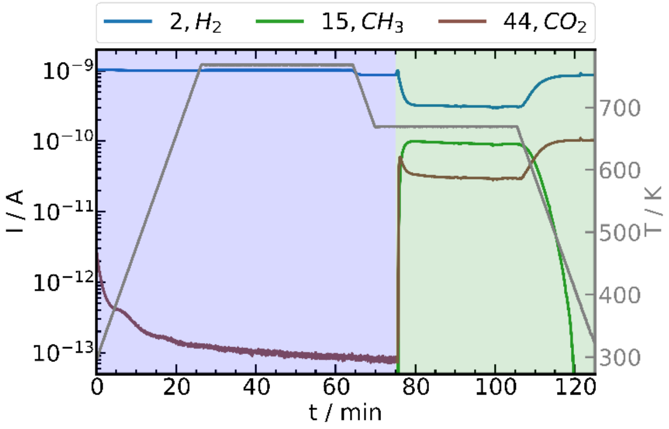

Figure S1 Selected mass spectrometry data of the microreactor experiments of the active Ni/Al_2O_3-ma sample with highlighted regions of activation (blue) and reaction (green) conditions (X_{CO2} ≈ 70 %) and the temperature (gray).

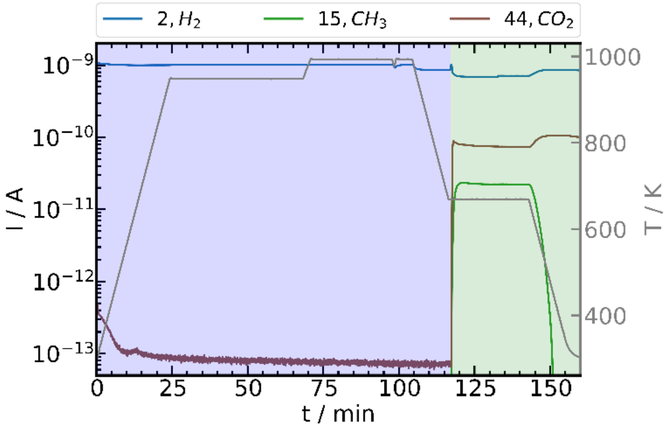

Figure S2 Selected mass spectrometry data of the microreactor experiments of the active Ni/Al_2O_3-ha sample with highlighted regions of activation (blue) and reaction (green) conditions (X_{CO2} ≈ 30 %) and the temperature (gray).

2. Ptychographic X-ray Computed Tomography Studies

2.1. Focused Ion Beam (FIB) Sample Preparation

Particles of the samples Ni/Al_2O_3-ma, Ni/Al_2O_3-mc, Ni/Al_2O_3-ha and Ni/Al_2O_3-hc after treatment in the microreactor were mounted on aluminum pins of the OMNY design[6] using a Strata 400 S FIB/SEM (FEI Company). A suitable particle was first attached to a micromanipulator with Pt and then transferred to the aluminum pins and fixed there with Pt. SEM images of the mounted particles of samples Ni/Al_2O_3-ma, Ni/Al_2O_3-mc, Ni/Al_2O_3-ha and Ni/Al_2O_3-hc are shown in Figure S3 and Figure S4, respectively. The particles are in size ranges of about 25 to 50 µm diameter. We picked particles and avoided extensive FIB cutting in order to reduce possible Ga contamination and ablation of the sample exterior, both of which can occur during extended FIB preparation procedures. This contamination could influence the electron density of the material in the PXCT experiments. It should be further noted that the contrast in the SEM images seems to be enhanced for the artificially coked samples compared to the activated samples. This might indicate the formation of graphitic carbon species on the outermost surface, which are more conductive compared to the pure catalysts. However, the images are not taken under exact same conditions, thus this is only interpreted as an anecdotal indication.

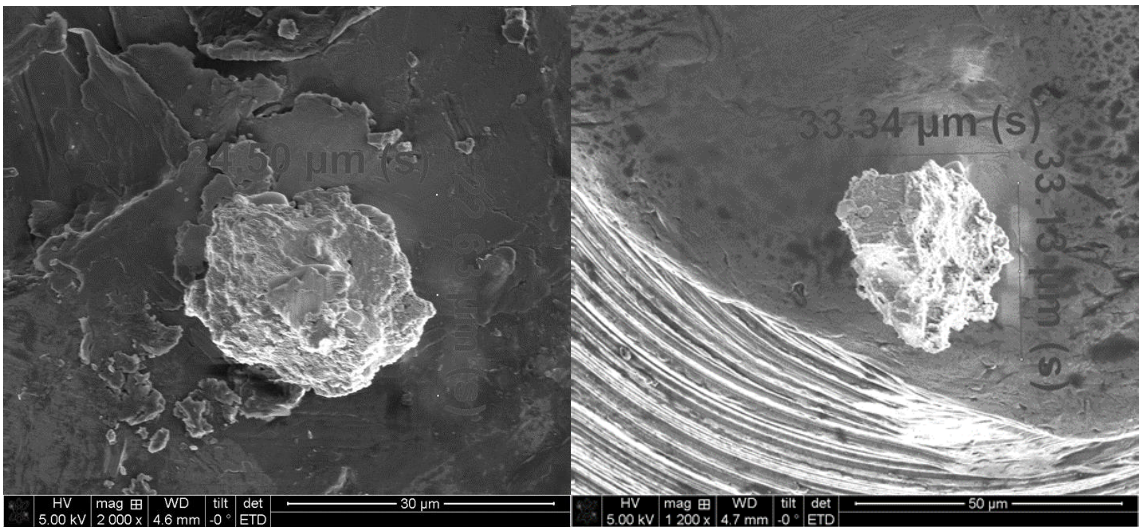

Figure S3 SEM images of the active Ni/Al_2O_3-ma (left) and coked Ni/Al_2O_3-mc (right) samples mounted on the Al-pin for the PXCT experiment with particle dimensions measured in the SEM.

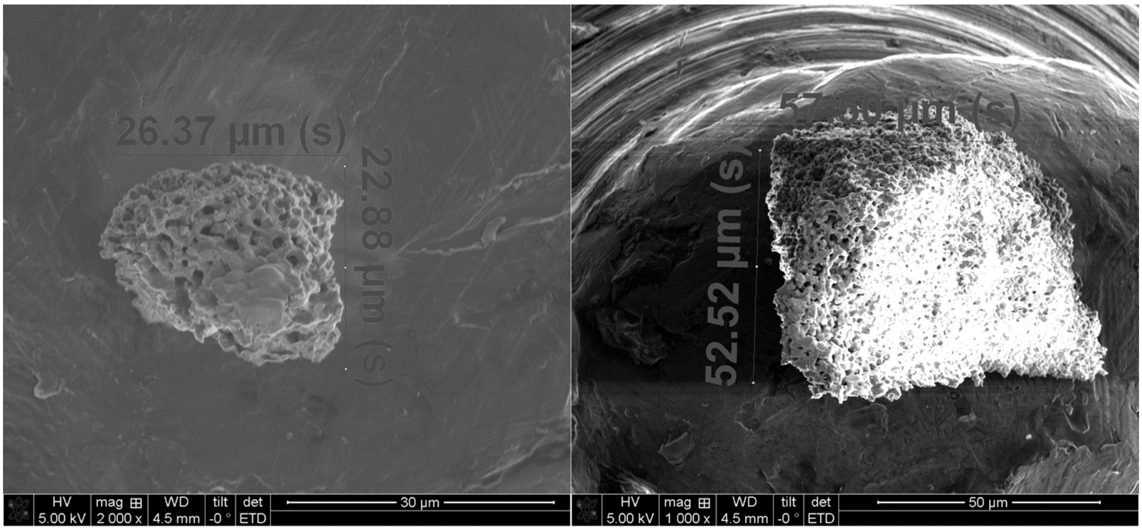

Figure S4 SEM images of the active Ni/Al_2O_3-ha (left) and coked Ni/Al_2O_3-hc (right) samples mounted on the Al-pins for the PXCT experiment with particle dimensions measured in the SEM. Left image reproduced from ref. [2].

2.2. X-ray Ptychography Experiments

Ptychographic X-ray computed tomography (PXCT) studies were performed at the coherence branch I13-1 beamline of the Diamond Light Source (Oxford, UK).[7,8] The experiments were carried out at a photon energy of 9.7 keV with a beam size of 5 µm diameter on the sample. The far-field diffraction patterns were recorded with a MerlinX detector with a Quad system of 512 x 512 pixels (pixel size 55 µm) at a sample to detector distance of 4.13 m. For the measurement of each tomogram, 1000 2D projections over an angular range of 180 ° (0.18 °/step) were recorded with different fields of view as summarized in Table S1. The total acquisition time for the samples ranged from 11 h to 19 h depending on the field of view. Ptychographic reconstructions with 400 iterations were done with the PtyREX package[9] based on the ePIE algorithm[10] to obtain a 2D projection series of the complex object transmission function, which contains information about the amplitude and phase shift. The reconstructions were performed on a cropped detector area of 256 x 256 pixels, which corresponds to a pixel size of 37.5 nm in the reconstructed projections. No modes, no upsampling and no position correction were performed.

Table S1 Measurement parameters for the PXCT experiments on the four Ni/Al_2O_3 samples.

Sample	Ni/Al_2O_3-ma	Ni/Al_2O_3-mc	Ni/Al_2O_3-ha	Ni/Al_2O_3-hc
Field of view (XY) / μm^2	33 x 16	38 x 12	40 x 22	54 x 16
Step size X / µm	1	1	1	1
Step size Y / µm	1	1	1	1
Exposure time / ms $step^{-1}$	30	30	30	30
Angular range / °	180	180	180	180
No. projections	1000	1000	1000	1000
Total acquisition time / h	12	11	19	18

2.3. Tomographic Reconstruction

The obtained 2D-projection series were aligned and tomographically reconstructed using the MATLAB script provided by Odstrčil *et al*.[11,12] The projection series was aligned by the following steps: (i) cross-correlation, (ii) vertical alignment based on mass fluctuation and (iii) tomographic consistency alignment[13] as described in ref.[12]. The tomograms were reconstructed using a filtered-back projection algorithm implemented in ref.[12] with a Ram-Lak filter. The obtained tomograms contain information about the complex refractive index *δ(r)*, from which the electron density N_e can be calculated as described in refs.[14,15] and shown in equation (3). The obtained N_e was offset individually for all samples to the mean value of air in close proximity to the particle. More details regarding the offset are provided in the Image Analysis section 2.5. The resolution of the obtained tomograms was estimated by 3D Fourier shell correlation (FSC).[12,16] For FSC the full dataset was divided in half to obtain two independent subtomograms with double angular spacing, which were then reconstructed individually. The FSC was then performed on the two reconstructed subtomograms using the ½ bit criterion as a threshold.[16] The FSC curves are shown for the samples in Figure S5, Figure S6, Figure S7 and Figure S8. The estimated resolution ranges from 74 nm to 83 nm.

It should be noted that in the Ni/Al_2O_3-m system some phase vortices artifacts are present, which are indicated by line features/streaks, e.g. N_e tomogram slice in Figure S10b. The artifacts may influence the alignment of the projection series and the quantitativeness of the N_e. Therefore, the Ni/Al_2O_3-m system is only presented here in the ESI and discussed as supporting indication to the results of the Ni/Al_2O_3-h system presented in the main manuscript. In case these artifacts are only caused by non-convergence of the ptychographic reconstruction and not due to physical reasons, a recently reported "VortRem" algorithm[17] or strategies as reported in ref. [18,19] or in the Toupy package[20] might be able to remove them. The origin of those artifacts might be pronounced edge like features or the strong absorbing residues of the Pt-gluing, which cannot be prevented in the chosen sample preparation by FIB-SEM.

The reconstructed *δ(r)* contrast tomograms are available under:

DOI: https://doi.org/10.5445/IR/1000132478 (Ni/Al_2O_3-ma)

DOI: https://doi.org/10.5445/IR/1000132479 (Ni/Al_2O_3-mc)

DOI: https://doi.org/10.5445/IR/1000132480 (Ni/Al_2O_3-ha)

DOI: https://doi.org/10.5445/IR/1000132481 (Ni/Al_2O_3-hc)

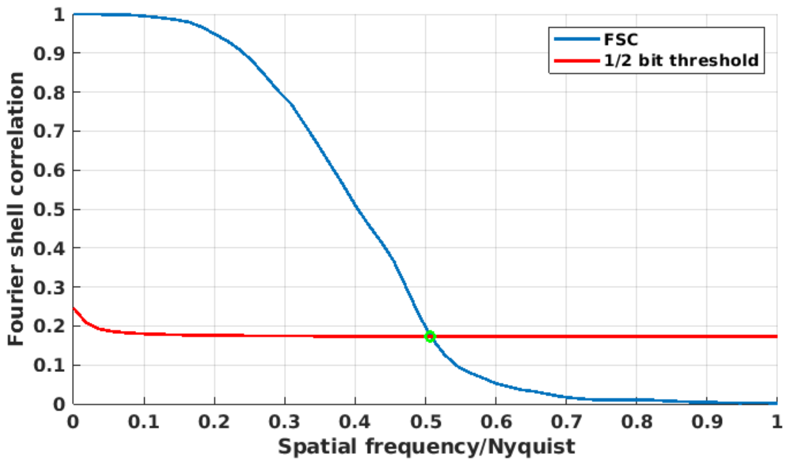

Figure S5 FSC analysis of the $NiAl_2O_3$-ma PXCT, the reconstructed pixel size is 37.5 nm and the resolution from the FSC is estimated as 74 nm.

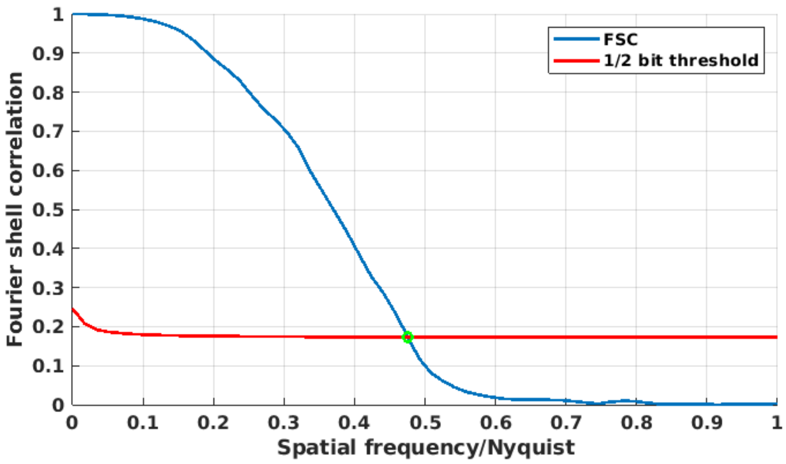

Figure S6 FSC analysis of the $NiAl_2O_3$-mc PXCT, the reconstructed pixel size is 37.5 nm and the resolution from the FSC is estimated as 79 nm.

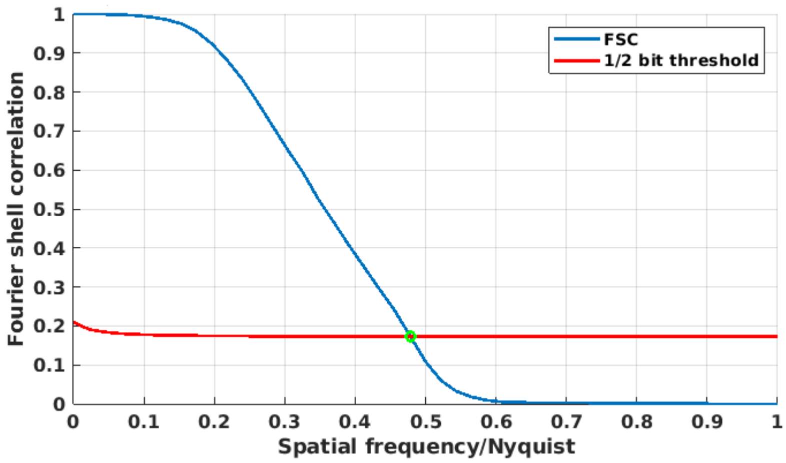

Figure S7 FSC analysis of the $NiAl_2O_3$-ha PXCT, the reconstructed pixel size is 37.5 nm and the resolution from the FSC is estimated as 78 nm.

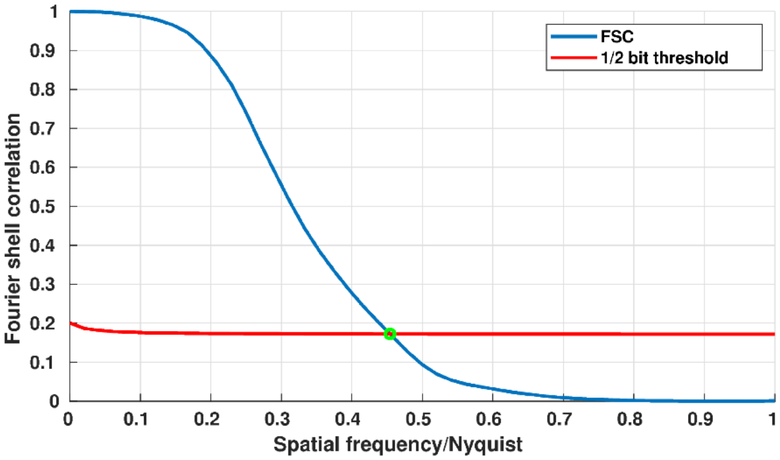

Figure S8 FSC analysis of the $NiAl_2O_3$*-hc PXCT, the reconstructed pixel size is 37.5 nm and the resolution from the FSC is estimated as 83 nm.*

2.4. Electron Density Reference

The reliability of the N_e information was checked with a 2D projection of a Siemens star reference sample (Figure S9). The Siemens star is a commonly used resolution target, in this case an Au structure of thickness of 225 nm. An area inside and outside the spoke with the same size was used to calculate a phase shift ϕ of –0.351 rad as shown in Figure S9. From ϕ the refractive index (δ) was calculated via the following equation:

$$\delta = -\phi \cdot \frac{\lambda}{2\pi \cdot \Delta d} \quad (2)$$

With λ as the wavelength and Δd as the thickness of the Au structure. From δ the electron density N_e can be calculated by the following relation:

$$N_e = \frac{2\pi}{\lambda^2 \cdot r_e} \cdot \delta \quad (3)$$

With r_e as the classic electron radius. This relation is valid away from absorption edges, which is the case for the elements present in our samples (Ni, Al, C, O).[14,15,21] One approximation of theoretical electron density can be calculated as:

$$N_e = \frac{\rho \cdot N_A \cdot f_1}{M} \quad (4)$$

With ρ as the mass density, N_A the Avogadro number, f_1 as the atomic scattering factor and M as the molar mass. The results for the experimental and theoretical N_e of the Au structure within the Siemens star and all parameters are summarized in Table S2. The experimental N_e of 4.33 $e^- Å^{-3}$ is in good agreement with the theoretical value of 4.39 $e^- Å^{-3}$ for the 225 nm thick gold structure of the Siemens star, which confirms the reliability of the electron density values obtained in the experiments.

Table S2 Parameters and electron density for the Siemens star reference sample.

Parameter	
λ / m	$0.128 \cdot 10^{-9}$
ϕ / rad	–0.351
Δd / m	$2.25 \cdot 10^{-7}$
δ / a.u.	$3.178 \cdot 10^{-5}$
r_e / m	$2.81794 \cdot 10^{-15}$
ρ / g cm^{-3}	19.3
M / g mol^{-1}	197
f_1 (at 9.7 keV)/ a.u.	74.4
N_e (experimental) / $e^- Å^{-3}$	4.33
N_e (theoretical) / $e^- Å^{-3}$	4.39

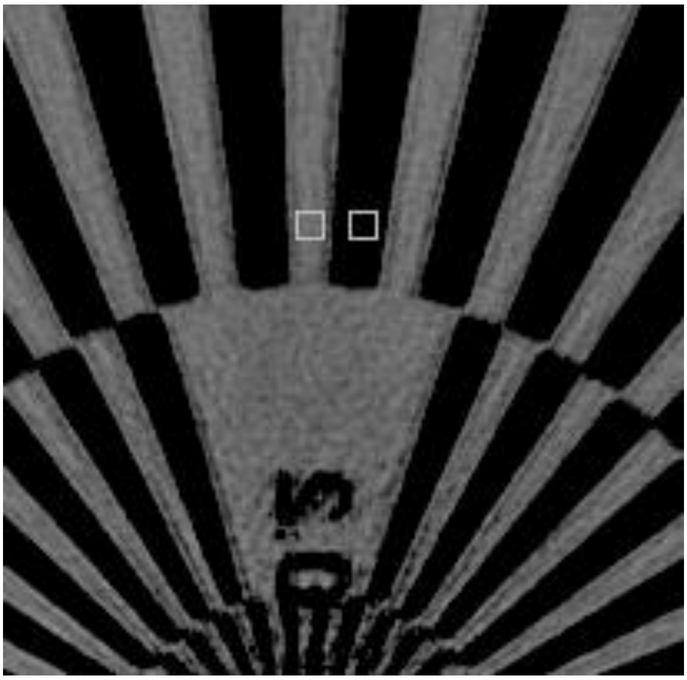

Figure S9 Analysis of the phase shift induced by the Au structure of the Siemens star reference material. The highlighted areas with same size were used to calculate the phase shift between air (bright) and gold (dark).

2.5. Image Analysis

The image analysis of all obtained tomograms was performed using Avizo 9.7.0 software (FEI SAS, Thermo Fisher Scientific). The analysis for both systems was performed identically despite the present artifacts in Ni/Al_2O_3-m. The following steps were carried out similarly for all samples. First the particle was masked from the surroundings using the *Lasso* and *Interpolation* tools. With that a label for the whole measured particle was obtained, which we refer to as the '*masked particle volume*'. Subsequently, we segmented the '*masked particle volume*' into '*catalyst body*', '*pore*' and '*contamination*' labels based on thresholding. The contamination observed is attributed to Ga or Pt deposition during the sample preparation for PXCT and is quite localized at certain positions of the particle exterior. The volume rendering of the '*contamination*' labels for the Ni/Al_2O_3-m and the Ni/Al_2O_3-h samples are shown in Figure S11 and Figure S19 and the '*catalyst body*' labels in Figure S12 and Figure S20, respectively. Additionally, for Ni/Al_2O_3-ma and Ni/Al_2O_3-mc the '*masked particle volume*', a cut through the volume showing the '*catalyst body*', '*contamination*' and '*pore*' labels are shown in Figure S10a,b, respectively.

The segmented '*masked particle volume*' and '*catalyst body*' labels are further used to analyze the N_e distribution in the tomograms. The '*masked particle volume*' was increased by 10 times applying the '*growth selection*' function of the segmentation editor to further mask out influence of the material. With another 10 times '*growth selection*' an air shell around the '*particle*' label with a thickness of about 300 nm (ca. 10 voxel) was selected. For this shell the N_e histogram of the surrounding air was computed and the N_e offset was determined from the maximum of the histogram as summarized for each sample in Table S3. The offset was then applied to the N_e. The resulting N_e histograms of the complete tomograms after offset are shown in Figure S14 and Figure S21 for Ni/Al_2O_3-m and Ni/Al_2O_3-h, respectively. These tomograms were used for further analysis.

To obtain spatially-resolved information about N_e, first the slice dependent histogram of N_e was computed for the '*masked particle volume*'. The mean N_e and the corresponding variance σ^2 are shown in Figure S15 and Figure S22 for Ni/Al_2O_3-m and Ni/Al_2O_3-h, respectively. One can clearly identify in both cases pronounced changes of the mean N_e starting from slice number 200. These pronounced changes can be attributed to larger parts of contamination due to Pt, which originates from sample mounting on the measurement pins. Therefore, a slice range from 1 to 200 was selected for further analysis of N_e and slices with contaminant were discarded.

The mean N_e with its variance σ^2 and standard deviation σ of the '*catalyst body*' label is shown in the main article Figure 3b for the selected slice range for Ni/Al_2O_3-h and in Figure S13 for Ni/Al_2O_3-m. Furthermore, in Figure S17 and Figure S24 the mean and mode N_e with the standard deviation σ can be found. The analysis of mean and mode is important, especially for the Ni/Al_2O_3-h system, where no distinct shift of the mode of the global N_e from the Ni/Al_2O_3-ha to the Ni/Al_2O_3-hc sample could be observed. The slice dependent analysis and consideration of the standard deviation can unravel the coking induced changes, by increased contribution at higher N_e.

For a thresholding based segmentation into coked and active parts of the '*catalyst body*' the N_e was normalized for the '*catalyst body*' label as shown in Figure S16 and Figure S23 for Ni/Al_2O_3-m and Ni/Al_2O_3-h, respectively. For Ni/Al_2O_3-m the maximum of the Ni/Al_2O_3-mc sample was used as segmentation threshold, while for Ni/Al_2O_3-h the point at higher N_e from which the distributions differed was chosen as the segmentation threshold. The thresholds are summarized in Table S3. This threshold-based segmentation is prone to errors, which is the reason we do not discuss these results quantitatively.

The potential errors of the segmentation were checked by propagating the N_e threshold to higher N_e values for the activated and coked samples as shown in Figure S18 and Figure S25 for Ni/Al_2O_3-m and Ni/Al_2O_3-h, respectively. For both systems we can also observe more electron dense material in the activated samples, which is due to the Gaussian shaped N_e distribution. However, in both cases the amount of coked labeled '*catalyst body*' is higher for the coked sample. For Ni/Al_2O_3-m (Figure S18) this difference is more pronounced as for Ni/Al_2O_3-h (Figure S25). The activated samples (Figure S18a and Figure S25a) also show a gradient of the N_e from the outside to the inside of the catalyst particle, which can be related to surface effects, i.e. more dense material at the outside than the inside. Nevertheless, for the coked samples (Figure S18b and Figure S25b) the gradient is much stronger, and much more of the inner material is affected, as well as the outer parts at higher thresholds. Based on this propagation analysis we infer that our conclusion that the coking starts in both systems in mesopores of the catalyst from the outside to the inside of the particles is reliable. Again, the comparison between Ni/Al_2O_3-m and Ni/Al_2O_3-h has to be taken carefully due to the present artifacts in Ni/Al_2O_3-mc. However, both systems show a similar behavior and trend that coking is more pronounced at the particle exterior. Furthermore, the coking is additionally proven by the Raman spectroscopy experiments for both systems (main paper, Figure 1).

Plotting of the results was carried out with self-written Python scripts using the matplotlib package (v3.4.1).[22]

Table S3 N_e offset values obtained from air in close proximity to the sample and the segmentation threshold to obtain the coked and activated catalyst body labels in Figure 3c,f of the main article. Difference values for the slice dependent analysis of the mean N_e of the catalyst body label of the activated and coked samples as shown in Figure 3b,e.

	Ni/Al_2O_3-ma	Ni/Al_2O_3-mc	Ni/Al_2O_3-ha	Ni/Al_2O_3-hc
N_e offset / e$^-$ Å^{-3}	+0.05	+0.075	+0.05	+0.055
N_e threshold segmentation / e$^-$ Å^{-3}		0.425		0.525
Difference N_e (coked) - N_e (activated)				
Mean difference / e$^-$ Å^{-3}	0.075		0.021	
Max. difference / e$^-$ Å^{-3}	0.101		0.038	
Min. difference / e$^-$ Å^{-3}	0.036		0.001	

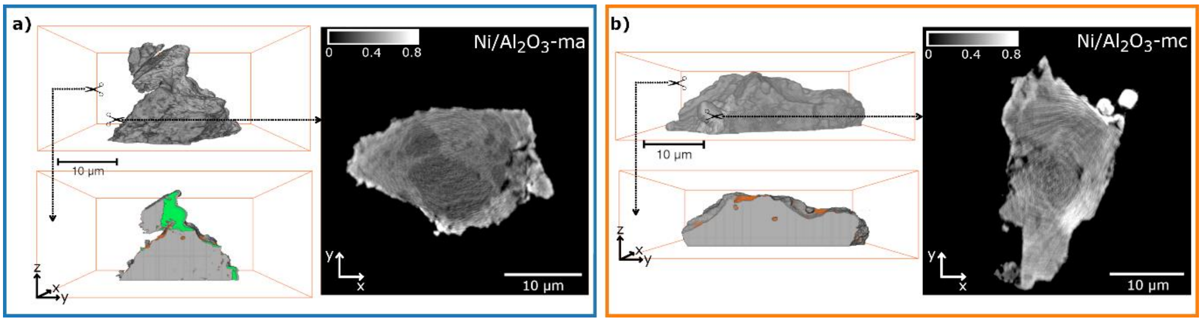

Figure S10 PXCT of activated catalyst Ni/Al_2O_3-ma (a) and artificial coked catalyst Ni/Al_2O_3-mc (b). Each reconstructed volume (gray) is shown with a cut through the middle, illustrating the segmented and labeled tomograms (gray = nanoporous catalyst body, orange = pores, green = contamination) and a grayscale N_e image of a typical slice through the volume (color bar in N_e / e$^-$ Å^{-3}, N_e offset to 0 with respect to air close to the sample, see Table S3).

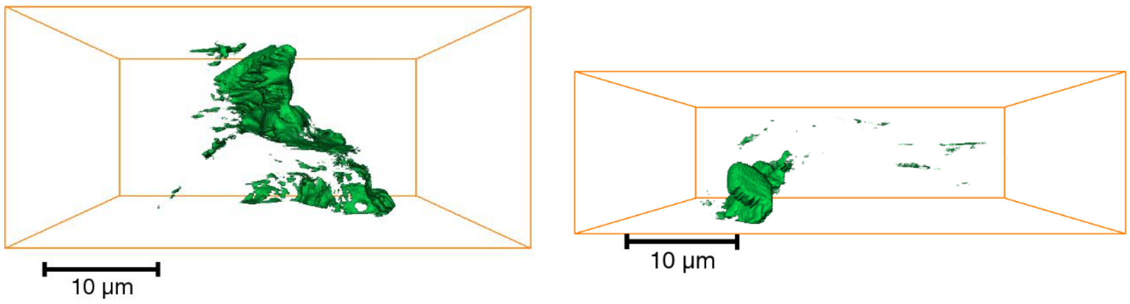

Figure S11 Segmented Pt contamination from FIB sample preparation for $NiAl_2O_3$-ma (left) and $NiAl_2O_3$-mc (right).

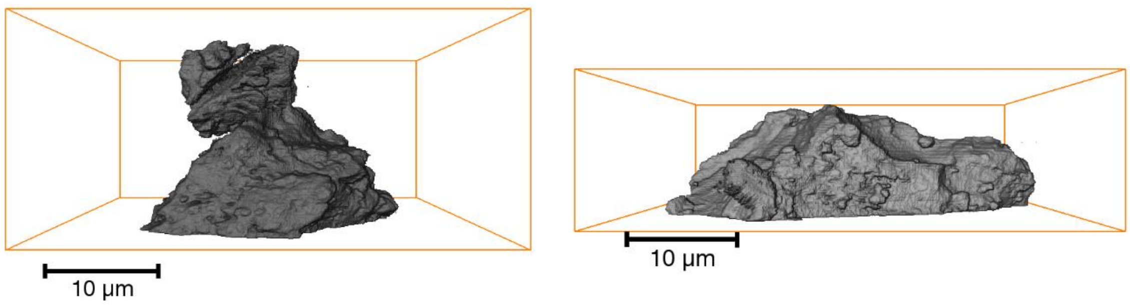

Figure S12 Segmented catalyst body label for $NiAl_2O_3$-ma (left) and $NiAl_2O_3$-mc (right).

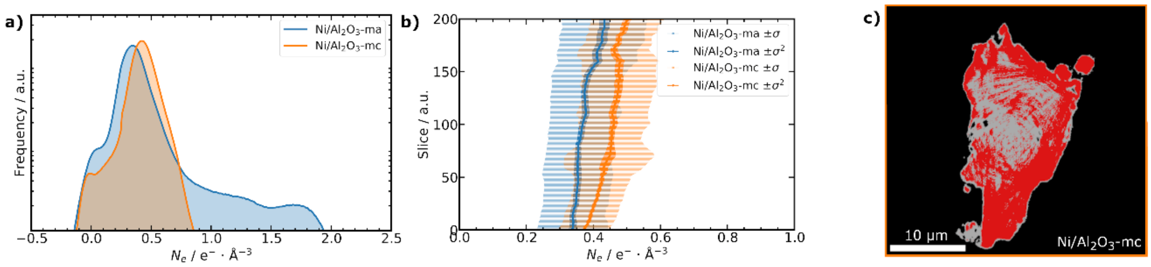

Figure S13 Global electron density distribution from PXCT of the activated and artificially coked catalyst particle labels of Ni/Al_2O_3-m (a). Mean, standard deviation (σ) and variance (σ^2) in N_e of the segmented catalyst body label in a selected slice range (b). Segmented tomogram slice of Ni/Al_2O_3-mc (c) showing less electron dense (gray, assigned to activated catalyst body) and higher density (red, assigned to coked catalysts body) regions by binary thresholding of the normalized N_e distribution (Figure S16). The reliability of the thresholding-based segmentation was tested for different thresholds (Figure S18)

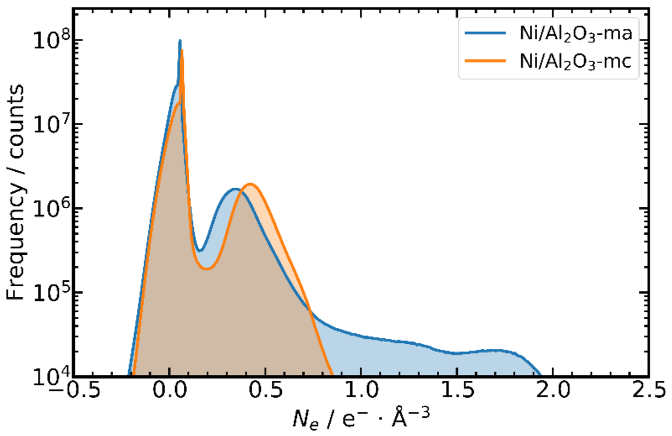

Figure S14 Total electron density distribution for the PXCT of samples $NiAl_2O_3$-ma and $NiAl_2O_3$-mc.

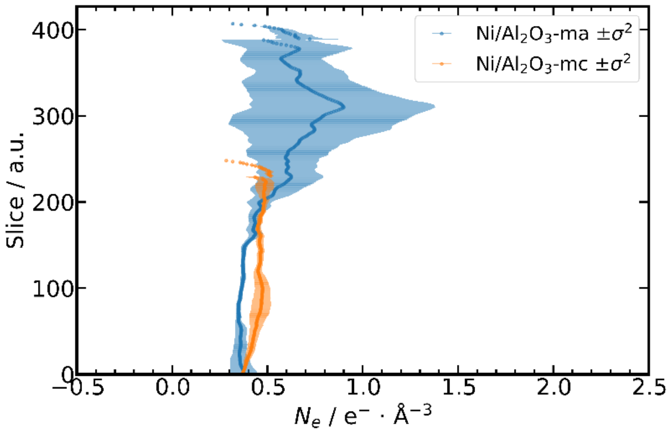

Figure S15 Mean electron density with the variance σ^2 for single slices of the masked particle volume of the PXCT of samples $NiAl_2O_3$-ma and $NiAl_2O_3$-mc.

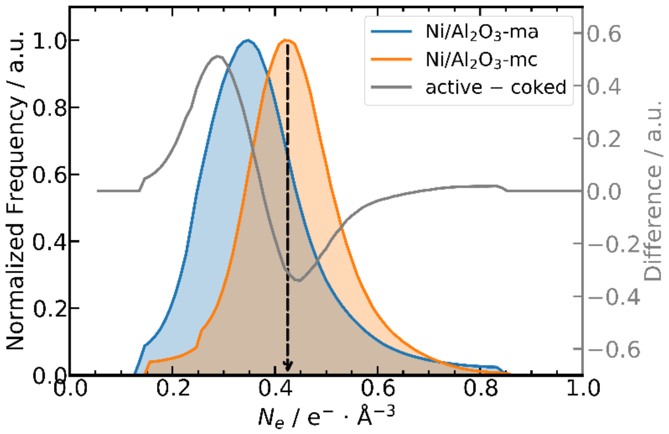

Figure S16 Normalized electron density distribution of the masked catalyst body label volume of the PXCT of samples $NiAl_2O_3$-ma and $NiAl_2O_3$-mc together with the difference curve. The arrow indicates the intersection used as threshold for segmentation in Figure 3e.

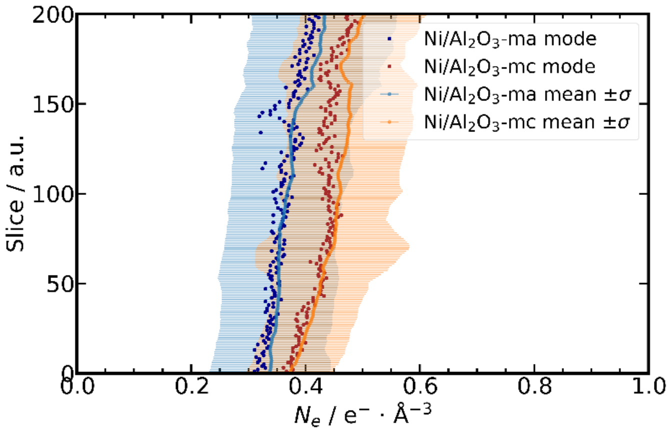

Figure S17 Comparison of the mean electron density with the standard deviation σ and mode of the electron density for single slices of the masked catalyst body label of the PXCT of samples NiAl$_2$O$_3$-ma and NiAl$_2$O$_3$-mc.

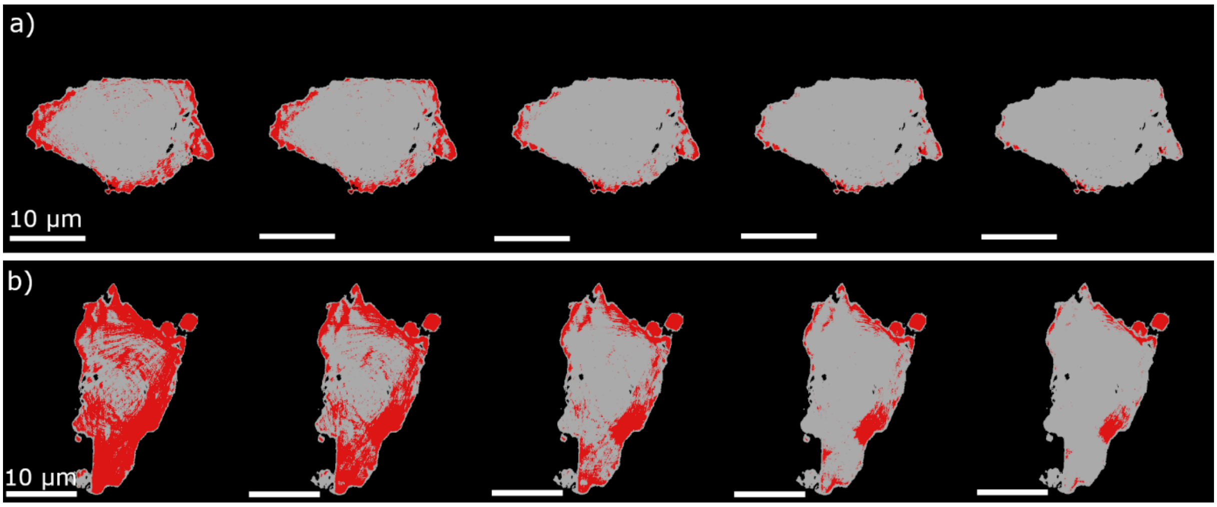

Figure S18 Propagation of the N_e threshold used for binary segmentation into activated (gray) and coked (red) catalyst body labels for (a) NiAl$_2$O$_3$-ma and (b) NiAl$_2$O$_3$-mc. N_e thresholds from left to right: 0.425, 0.475, 0.525, 0.575, 0.625 e$^-$ Å^{-3}. Scale bar 10 µm.

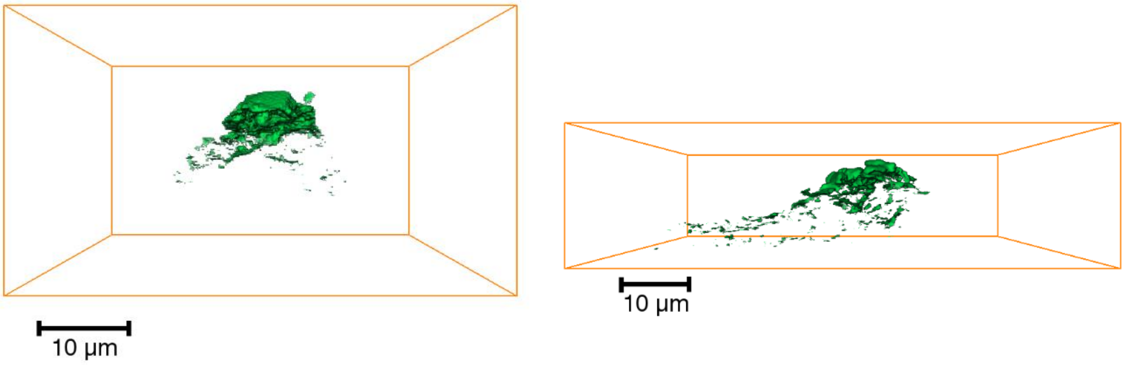

Figure S19 Segmented Pt contamination from FIB sample preparation for NiAl$_2$O$_3$-ha (left) and NiAl$_2$O$_3$-hc (right).

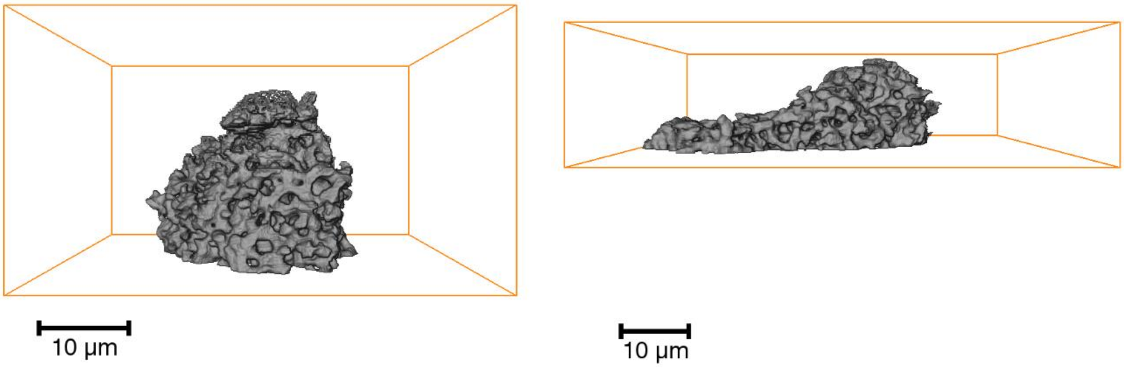

Figure S20 Segmented catalyst body label for $NiAl_2O_3$-ha (left) and $NiAl_2O_3$-hc (right).

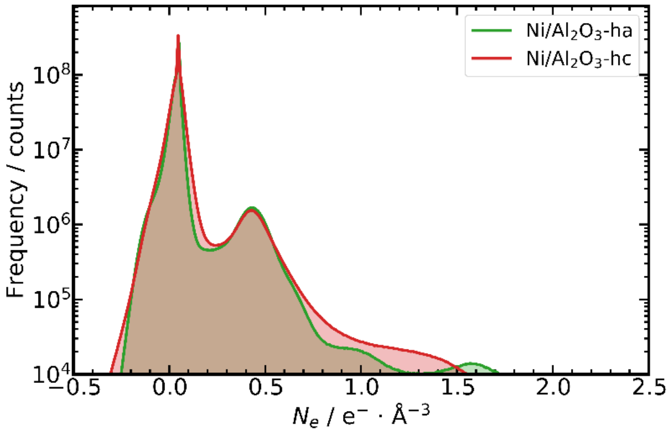

Figure S21 Total electron density distribution for the PXCT of samples $NiAl_2O_3$-ha and $NiAl_2O_3$-hc.

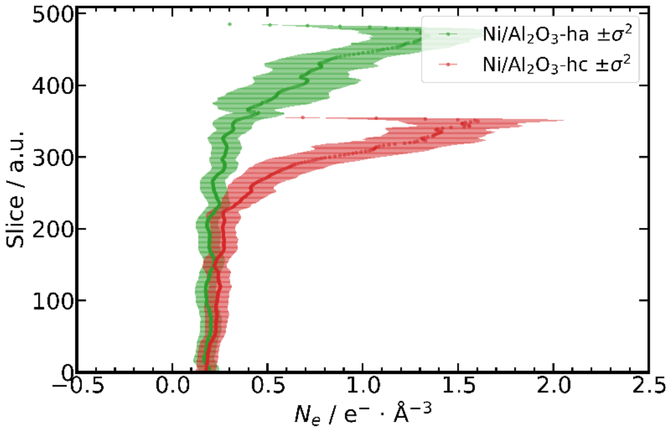

Figure S22 Mean electron density with the variance σ^2 for single slices of the masked particle volume of the PXCT of samples $NiAl_2O_3$-ha and $NiAl_2O_3$-hc.

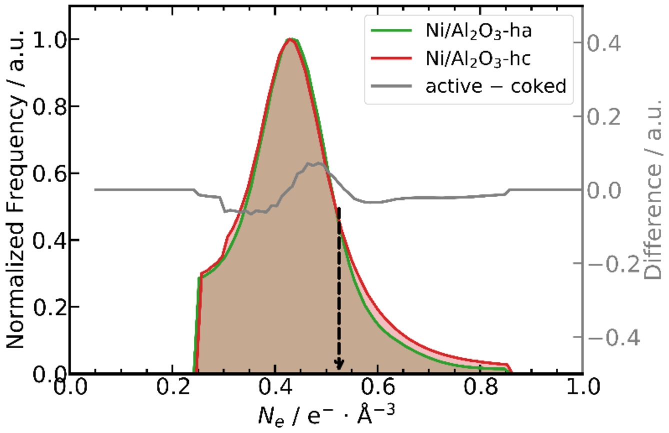

Figure S23 Normalized electron density distribution of the masked catalyst body label volume of the PXCT of samples $NiAl_2O_3$-ha and $NiAl_2O_3$-hc together with the difference curve. The arrow indicates the intersection used as threshold for segmentation in Figure 3e.

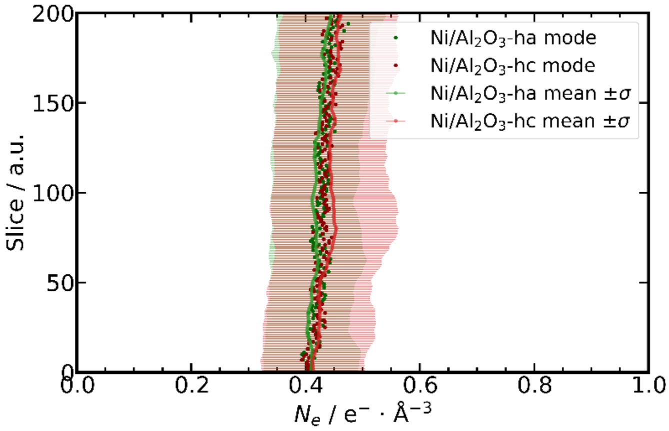

Figure S24 Comparison of the mean electron density with the standard deviation σ and mode of the electron density for single slices of the masked catalyst body label of the PXCT of samples $NiAl_2O_3$-ha and $NiAl_2O_3$-hc.

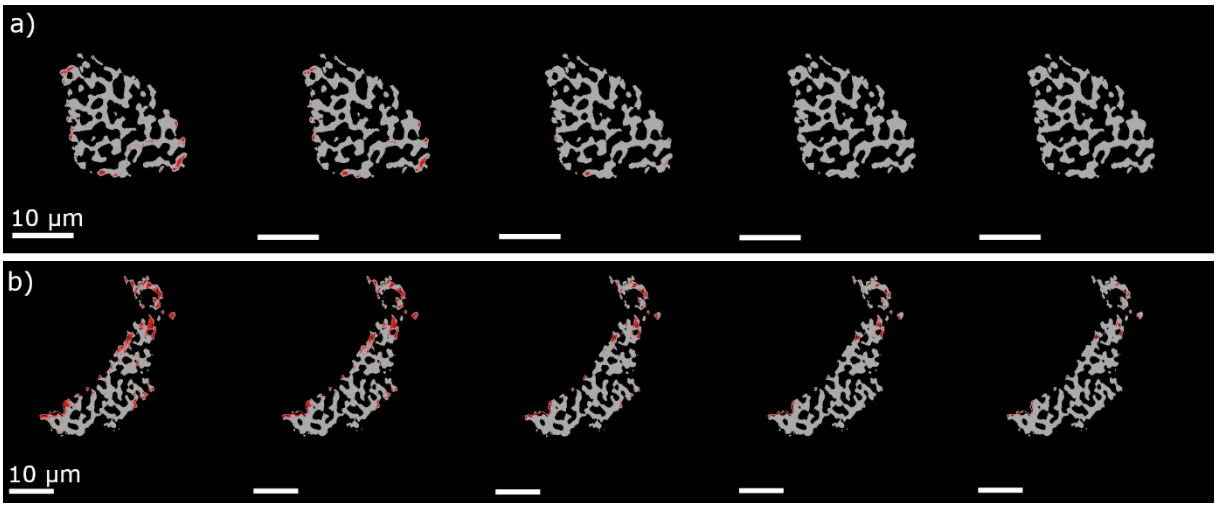

Figure S25 Propagation of the N_e threshold used for binary segmentation into activated (gray) and coked (red) catalyst body labels for (a) $NiAl_2O_3$-ha and (b) $NiAl_2O_3$-hc. N_e thresholds from left to right: 0.525, 0.555, 0.605, 0.655, 0.705 $e^- Å^{-3}$. Scale bar 10 µm.

References

[1] B. Mutz, M. Belimov, W. Wang, P. Sprenger, M.-A. Serrer, D. Wang, P. Pfeifer, W. Kleist, J.-D. Grunwaldt, *ACS Catal.* **2017**, *7*, 6802–6814.

[2] S. Weber, K. L. Abel, R. T. Zimmermann, X. Huang, J. Bremer, L. K. Rihko-Struckmann, D. Batey, S. Cipiccia, J. Titus, D. Poppitz, C. Kübel, K. Sundmacher, R. Gläser, T. L. Sheppard, *Catalysts* **2020**, *10*, 1–22.

[3] J. Herwig, J. Titus, J. Kullmann, N. Wilde, T. Hahn, R. Gläser, D. Enke, *ACS Omega* **2018**, *3*, 1201–1212.

[4] J.-D. Grunwaldt, M. Caravati, S. Hannemann, A. Baiker, *Phys. Chem. Chem. Phys.* **2004**, *6*, 3037–3047.

[5] B. Mutz, P. Sprenger, W. Wang, D. Wang, W. Kleist, J.-D. Grunwaldt, *Appl. Catal. A Gen.* **2018**, *556*, 160–171.

[6] M. Holler, J. Raabe, R. Wepf, S. H. Shahmoradian, A. Diaz, B. Sarafimov, T. Lachat, H. Walther, M. Vitins, *Rev. Sci. Instrum.* **2017**, *88*, 113701.

[7] C. Rau, D. Batey, S. Cipiccia, X. Shi, S. Marathe, M. Storm, A. J. Bodey, M.-C. Zdora, *Proc. SPIE* **2019**, 31.

[8] D. Batey, S. Cipiccia, X. Shi, S. Williams, K. Wanelik, A. Wilson, S. Pérez-Tamarit, P. Cimavilla, M. A. Ródriguez-Pérez, C. Rau, *Microsc. Microanal.* **2018**, *24*, 42–43.

[9] D. Batey, Ptychographic Imaging of Mixed States, University of Shefffield, **2014**.

[10] A. M. Maiden, J. M. Rodenburg, *Ultramicroscopy* **2009**, *109*, 1256–1262.

[11] M. Guizar-Sicairos, A. Diaz, M. Holler, M. S. Lucas, A. Menzel, R. A. Wepf, O. Bunk, *Opt. Express* **2011**, *19*, 21345.

[12] M. Odstrčil, M. Holler, J. Raabe, M. Guizar-Sicairos, *Opt. Express* **2019**, *27*, 36637.

[13] M. Guizar-Sicairos, J. J. Boon, K. Mader, A. Diaz, A. Menzel, O. Bunk, *Optica* **2015**, *2*, 259.

[14] M. Dierolf, A. Menzel, P. Thibault, P. Schneider, C. M. Kewish, R. Wepf, O. Bunk, F. Pfeiffer, *Nature* **2010**, *467*, 436–439.

[15] A. Diaz, P. Trtik, M. Guizar-Sicairos, A. Menzel, P. Thibault, O. Bunk, *Phys. Rev. B - Condens. Matter Mater. Phys.* **2012**, *85*, 1–4.

[16] M. van Heel, M. Schatz, *J. Struct. Biol.* **2005**, *151*, 250–262.

[17] I. Zanette, R. Clare, D. Eastwood, C. Venkata, F. Pfeiffer, P. Cloetens, P. Thibault, *Phys. Rev. Appl.* **2020**, *14*, 1.

[18] J. C. da Silva, P. Trtik, A. Diaz, M. Holler, M. Guizar-Sicairos, J. Raabe, O. Bunk, A. Menzel, *Langmuir* **2015**, *31*, 3779–3783.

[19] A. Cuesta, A. G. De La Torre, I. Santacruz, P. Trtik, J. C. da Silva, A. Diaz, M. Holler, M. A. G. Aranda, *J. Phys. Chem. C* **2017**, *121*, 3044–3054.

[20] J. C. da Silva, "Toupy," can be found under https://toupy.readthedocs.io/en/latest/index.html, **2019**.

[21] F. Pfeiffer, *Nat. Photonics* **2018**, *12*, 9–17.

[22] J. D. Hunter, *Comput. Sci. Eng.* **2007**, *9*, 90–95.
